# Device-Related Thrombotic Microangiopathy in an Elderly Patient With a History of Aortic Surgery

**DOI:** 10.7759/cureus.27937

**Published:** 2022-08-12

**Authors:** Chihiro Tanaka, Yumi Naito, Shoichi Suehiro, Chiaki Sano, Ryuichi Ohta

**Affiliations:** 1 Family Medicine, Faculty of Medicine, Shimane University, Izumo, JPN; 2 Communiy Care, Unnan City Hospital, Unnan, JPN; 3 Cardiac Surgery, Faculty of Medicine, Shimane University, Izumo, JPN; 4 Community Medicine Management, Faculty of Medicine, Shimane University, Izumo, JPN

**Keywords:** general physician, rural hospitals, fragmented red blood cells, thrombocytopenia, device-related, atypical hemolytic uremic syndrome, thrombotic microangiopathy

## Abstract

Thrombotic microangiopathy (TMA) is caused by several diseases, including infections, congenital and autoimmune diseases, and malignancies, usually requiring admission to intensive care. The primary pathophysiology of TMA is microvascular thrombosis, and its diagnosis is based on the presence of hemolysis, thrombocytopenia, schistocytes in a blood smear, and organ damage. Among secondary TMAs, device-related TMA could be difficult to diagnose if device implementation was performed years ago. We report the case of an 87-year-old woman with a chief complaint of dyspnea diagnosed with device-related TMA. In device-related TMA, thrombogenesis/thrombocytopenia is triggered by hemolysis/fragmented red blood cells. However, in other TMAs, thrombogenesis or thrombocytopenia is preceded by hemolysis and the presence of fragmented red blood cells. Thus, rapid plasma exchange is necessary to address TMA pathogenesis. TMA can be managed in a community hospital if the facility has access to plasma exchange. It is possible to treat complex TMAs even in community hospitals by carefully considering their pathophysiology. Additionally, improving the quality of general practice in community hospitals will allow for more effective diagnosis and treatment of TMAs.

## Introduction

Thrombotic microangiopathy (TMA), a critical condition caused by several diseases, requires intensive treatment. Its pathophysiology is primarily thrombosis in the microcirculation. The diagnosis is based on the findings of hemolysis, thrombocytopenia, the presence of schistocytes in a blood smear, and organ damage [1]. Thrombotic thrombocytopenic purpura (TTP) is a critical diagnosis that requires emergency plasma exchange and immunosuppressive treatment [2]. TTP is divided into two types, congenital and acquired, and is differentiated by the presence of an inhibitor of ADAMTS-13 (a disintegrin and metalloproteinase with a thrombospondin type 1 motif, member 13). ADAMTS-13 is a metalloprotease that cleaves the von Willebrand factor (vWF), a large protein involved in blood clotting, and degrades large vWF multimers. The decrease in the activity of ADAMTS-13 causes the accumulation of ultra-high-molecular-weight (UHMW) vWF on endothelial cells, leading to thrombus formation on arterial walls [3]. Another disease resembling TMA is a hemolytic uremic syndrome caused by infection with *Escherichia coli* O157, a bacterium that releases ciguatoxins [1,3]. Supportive care using plasma exchange may be the mainstream treatment. Secondary TMA is caused by autoimmune diseases, medications, pregnancy, and device-related factors. It is treated by managing the primary condition [1,3].

Device-related TMA as a secondary TMA could be difficult to diagnose if device implementation was performed years ago. Previous reports have demonstrated that cardiac valve-related TMA can appear soon after cardiac valve surgery [4,5], where it was diagnosed by excluding other TMA possibilities [6]. Here, we report the case of an older woman with a chief complaint of dyspnea and the pathophysiology of secondary TMA. In addition, we discuss the diagnostic difficulty of valve-related TMA and practical methods to diagnose the disease in rural hospitals.

## Case presentation

**Figure 1:**
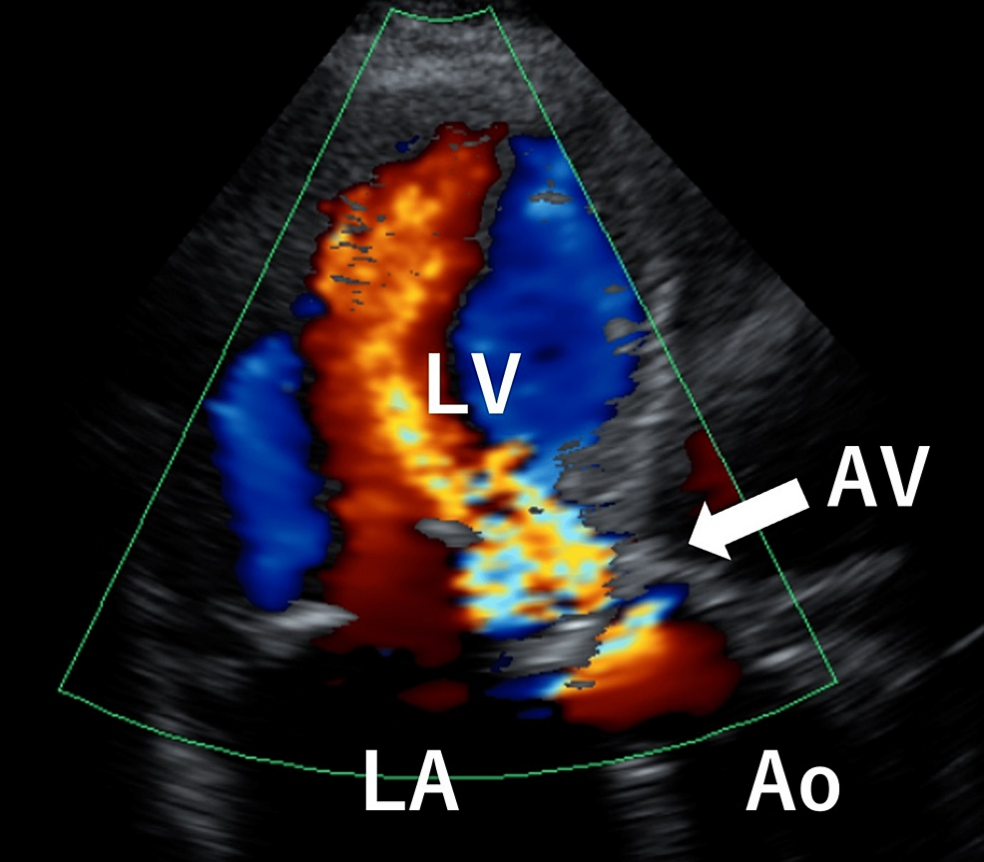
Echocardiography on admission showing severe aortic regurgitation (arrow). Ao, aorta; AV, aortic valve; LA, left atrium; LV, left ventricle.

An 87-year-old woman was admitted to our hospital with complaints of anterior chest discomfort, respiratory distress, and facial and lower leg edema. She had a history of repeated hospitalizations due to acute exacerbation of chronic heart failure. Thrombocytopenia, red blood cell fragmentation, and rapid liver and renal function deterioration were observed at admission. In 2013, she underwent surgery for aortic valve stenosis. The aortic valve was replaced with a prosthetic valve; however, the prosthetic valve was impaired with perivalvular leak eight years post-surgery. Thus, severe aortic regurgitation was observed using heart ultrasonography (Figure 1).

Other comorbidities included acute liver failure, chronic kidney disease, and sleep apnea. The patient was administered ferrous citrate (50 mg), tolvaptan (7.5 mg), rosuvastatin (2.5 mg), pramipexole (0.5 mg), and warfarin (0.5 mg). Her vital signs on admission were as follows: height, 150 cm; weight, 54.1 kg; temperature, 35.4°C; blood pressure, 115/85 mmHg; pulse, 110 beats/min; and oxygen saturation (SpO_2_), 98% (oxygen, 6 L). The patient had jugular vein distension, profuse sweating, edema of the face and lower legs, and chest wheezing. Hemorrhagic spots were observed in the conjunctiva, oral cavity, left chest, and lower limbs. There was no erythema or other external finding suggestive of collagen disease. Her blood pressure was normal, ruling out TMA due to hypertensive emergencies. Blood tests showed a low platelet count of 4.6 × 10^4^/μL and abnormal values of aspartate aminotransferase (AST, 427 IU/L), alanine aminotransferase (ALT, 350 IU/L), lactate dehydrogenase (LDH, 1,235 U/L), total bilirubin (6.3 mg/dL), direct bilirubin (2.4 mg/dL), blood urea nitrogen (BUN, 68.1 mg/dL), and creatinine (1.84 mg/dL). Abnormal coagulation values were observed with a prothrombin time (PT) of 1.2% and an international normalized ratio of prothrombin time (PT-INR) of <9.00. Although the direct and indirect Coombs test results were negative, her haptoglobin level was <10 mg/dL (Table 1).

**Table 1:** Initial laboratory data of the patient. PT, prothrombin time; PT-INR, international normalized ratio of prothrombin time; APTT, activated partial thromboplastin time; AT-III, antithrombin; vWF, von Willebrand factor; HBs, hepatitis B surface; CK, creatine kinase; CRP, C-reactive protein; KL-6, sialylated carbohydrate antigen KL-6; CCP antibody, anti-cyclic citrullinated peptide antibody; SS-A, Sjogren’s syndrome-A; SS-B, Sjogren’s syndrome-B; ADAMTS-13, a disintegrin and metalloproteinase with a thrombospondin type 1 motif, member 13; eGFR: estimated glomerular filtration rate; MPO-ANCA, myeloperoxidase-anti-neutrophil cytoplasmic antibodies; T.U., titer units.

Marker	Level	Reference
White blood cells	9.3	3.5-9.1 × 10^3^/μL
Neutrophils	78.4	44.0%-72.0%
Lymphocytes	14.0	18.0%-59.0%
Monocytes	7.4	0.0%-12.0%
Eosinophils	0.0	0.0%-10.0%
Basophils	0.2	0.0%-3.0%
Red blood cells	4.29	3.76-5.50 × 10^6^/μL
Hemoglobin	13.2	11.3-15.2 g/dL
Hematocrit	39.8	33.4%-44.9%
Mean corpuscular volume	92.7	79.0-100.0 fl
Platelets	4.6	13.0-36.9 × 10^4^/μL
PT	1.2	70%-130%
PT-INR	<9.00	
APTT	47.3	25-40 s
Fibrinogen	189.3	200-400 mg/dL
Fibrinogen degradation products	7.1	<5 μg/mL
D-dimer	1.80	～1.00 μg/mL
AT-Ⅲ activity	55.0	80%-120％
vWF activity	59	60%-170％
Total bilirubin	6.3	0.2-1.2 mg/dL
Direct bilirubin	2.4	0-0.4 mg/dL
Aspartate aminotransferase	427	8-38 IU/L
Alanine aminotransferase	350	4-43 IU/L
γ-Glutamyl transpeptidase	151	<48 IU/L
Lactate dehydrogenase	1235	121-245 U/L
Uric acid	11.1	3.0-6.9 mg/dL
Blood urea nitrogen	68.1	8-20 mg/dL
Creatinine	1.84	0.40-1.10 mg/dL
eGFR	20.4	>60.0 mL/min/L
Serum Na	134	135-150 mEq/L
Serum K	5.1	3.5-5.3 mEq/L
Serum Cl	98	98-110 mEq/L
Serum Ca	9.0	8.8-10.4 mg/dL
Ferritin	208.0	14.4-303.7 ng/mL
CK	157	56-244 U/L
CRP	0.51	<0.30 mg/dL
Coombs test		
Indirect Coombs	Negative	
Haptoglobin	≦10	mg/dL
ADAMTS-13		
ADAMTS-13 activity (IU/mL)	0.21	0.10 IU/mL
ADAMTS-13 activity (%)	21	10~
ADAMTS-13 inhibitor (BU/mL)	<0.5	～0.5 BU/mL
ADAMTS-13 inhibitor judgment	Negative	
Immunological tests		
HBs antigen	0.00	IU/mL
HBs antibody	0.00	mIU/mL
Syphilis	0.0	T.U.
Antinuclear antibody	40	Twice
Homogeneous	40	
Speckled	40	
Nucleolar	Negative	
Peripheral	Negative	
Discrete speckled	Negative	
Cytoplasm	Negative	
C3	51	86-160 mg/dL
C4	11	17-45 mg/dL
KL-6	388	105.3~401.2 U/mL
MPO-ANCA	<1.0	～3.5 U/mL
β-D-Glucan	7.1	～20.0 pg/mL
SS-A antibody	<1.0	～10.0 U/mL
SS-B antibody	<1.0	～10.0 U/mL
CCP antibody	0.8	U/mL
IgG4	18	11-121

**Figure 2:**
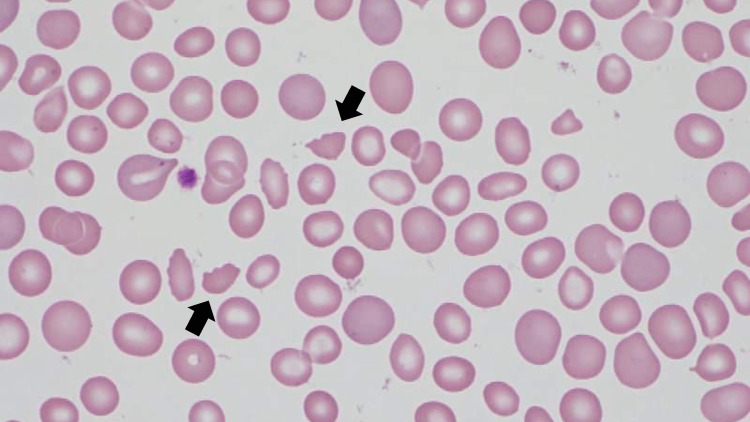
Fragmented red blood cells in the blood smear (black arrows).

The peripheral blood smear examination revealed fragmented erythrocytes of different sizes (Figure 2).

Serological tests for ADAMTS-13 activity and inhibitors were performed to classify TMA type. Noninvasive positive pressure ventilation and furosemide intravenous injection (20 mg) were administered to reduce the afterload due to acute exacerbation of heart failure and relieve her symptoms. As the possibility of TTP could not be ruled out and thrombocytopenia progressed, the patient was treated with two plasma exchanges and 1,000 mg of intravenous methylprednisolone for three days, followed by oral prednisolone 60 mg/day from the fourth day of admission. After the second plasma exchange, ADAMTS-13 activity was within the reference level, and the inhibitors were negative. Thus, TTP was ruled out, and plasma exchange was stopped.

Atypical hemolytic uremic syndrome (aHUS), secondary TMA, and device-related TMA remained the differential diagnoses. Complement-associated TMA was suspected because of decreased serum complement titers; however, the observation of decreased C4 levels was atypical and unlikely (Table 1). Therefore, we sought to differentiate secondary TMA. Immunological testing was negative for several autoimmune antibodies; thus, we considered TMA a complicated autoimmune disease. Although the possibility of malignancy could not be ruled out due to the high levels of LDH, it was considered unlikely because of the absence of gross lesions on computed tomography and her past normal upper and lower gastrointestinal endoscopy results.

On the ninth day of hospitalization, the patient began to have impaired consciousness, and we suspected sepsis due to pyelonephritis. Thus, we administered piperacillin and tazobactam as empirical therapy, which provided primary relief. *Enterococcus faecalis* and *E. coli* were isolated from urine samples, and ampicillin was administered. On the 18th day, the serum ammonia level was high (107 μg/mL). The patient was treated with lactulose and stool control therapies considering the possibility of ammonia encephalopathy.

On the 18th day of admission, the platelet count decreased again to 1.9 × 10^4^/µL. aHUS was suspected, and plasma exchange was performed again; however, the platelet count did not recover. On the 21st day of admission, considering device-related TMA, we improved congestive heart failure with reduced blood flow through the aortic valve, resulting in the platelet count recovery to 3.1 × 10^4^/μL. On the 26th day of admission, the patient suddenly became unconscious, hypotensive, and bradycardic. After talking to the patient’s family, the best supportive care was provided, after which the patient died.

## Discussion

This case report presents an example of TMA caused by the disruption of vascular endothelial cells. Fragmented red blood cells are formed by valve destruction following aortic valve surgery. Here, we discuss the importance of differentiating device-related TMA from other types of TMAs, the pathophysiology of device-related TMA, and the diversity of its pathological progression.

The differentiation of device-related TMA from other TMAs is based on the need for plasma exchange. Unexplained hemolytic anemia and thrombocytopenia are suspected causes of TMA and require testing for Shiga toxin-producing *E. coli* (STEC), which has high lethality rates and ADAMTS-13 activity. A positive STEC result indicates STEC-hemolytic uremic syndrome (HUS), and ADAMTS-13 activity of <10% indicates TTP [2,7]. However, because both tests take time to yield results, symptoms continue to develop, and platelet loss may become progressive. In such cases, it is necessary to perform plasma exchange without waiting for results, and caution should be employed [8].

Although it is relatively easy to differentiate between STEC-HUS and TTP, the differential diagnosis of other TMAs is often difficult; all of these diseases are diagnosed as aHUS [5,9]. It is now widely recognized that aHUS diseases are complement-associated TMA [9]. In patients with negative STEC and ADAMTS-13 activity >10%, secondary TMA is diagnosed in the presence of autoimmune diseases, hematopoietic stem cell transplantation, organ transplantation, malignancy, pregnancy, or drug use [9]. Currently, no specific diagnostic criteria are available for aHUS. Complement C3 and C4, anti-H factor antibodies, and hemolytic assays were tested for diagnosis; however, the clinical diagnosis was based on various findings [10]. Ultimately, genetic analysis of the complement factors confirmed the diagnosis of aHUS. Although low C3 and normal C4 levels were important findings, they were not observed in all cases. Clinically, a small number of cases without genetic abnormalities have been reported [10]. 

The diagnosis of device-associated TMA was made after ruling out the possibility of TMA. In the present case, ADAMTS-13 activity was >10%, TTP was ruled out, and an aHUS was initially diagnosed. Complement factor gene analysis showed decreased C3 and C4 levels, suggesting that the present condition is not complement-related TMA but complement activation due to vascular endothelial cell damage, resulting in consumptive complement loss [10]. In our case, device-related TMA was the most likely differential diagnosis after excluding other diseases. We treated the patient considering that hemolysis improved by reducing the blood flow through the heart valve affected by dehydration. Once the patient's symptoms were resolved with intensive treatment for heart failure, it was considered a surrogate for the diagnosis of device-related TMA.

**Figure 3:**
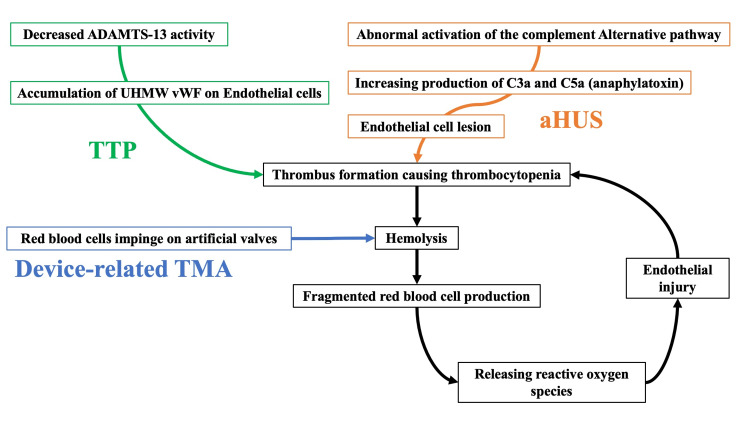
The difference between device-related TMA and other TMAs (TTP and aHUS). aHUS, atypical hemolytic uremic syndrome; TMA, thrombotic microangiopathy; TTP, thrombotic thrombocytopenic purpura; UHMW, ultra-high-molecular-weight; vWF, von Willebrand factor.

Device-associated TMA causes hemolysis and the production of fragmented red blood cells when red blood cells are corroded by an impaired artificial valve. Fragmented red blood cells release reactive oxygen species and cause oxidative stress, resulting in vascular endothelial injury [7]. Platelets adhere to and aggregate on collagen fibers exposed to endothelial damage, forming thrombi, which eventually cause thrombocytopenia [7]. Red blood cells collide with thrombi formed on small blood vessels, resulting in massive hemolysis and fragmented red blood cell formation [3]. The pathophysiological flow was the opposite in device-related TMA and other TMA (Figure 3).

In device-related TMA, hemolysis/fragmented red blood cell formation occurs first, causing thrombus formation and thrombocytopenia. However, in other TMAs, hemolysis/fragmented red blood cell formation is triggered after thrombus formation and thrombocytopenia. Our patient experienced hemolysis and fragmented red blood cells due to impingement on the prosthetic valve, which caused thrombus formation and thrombocytopenia as a negative spiral.

Here, we discuss the pathophysiology of device-related TMA by stabilizing circulation and cardiac-related controls. Rapid plasma exchange is important in response to TMA conditions. Community hospitals can manage TMA if their facilities perform plasma exchange. In addition, we believe that general physicians who can manage systemic conditions must recognize TMA's pathophysiology accurately. Physicians can provide prompt treatment by recognizing TMA as a differential diagnosis in patients with impaired consciousness and thrombocytopenia. It is necessary to provide education in general hospitals to increase awareness of the different pathophysiologies of TMA [11,12]. Treating complex TMA at a community hospital is possible by carefully considering its pathophysiology. In the future, it will be necessary to improve the quality of general practices in the community [13,14].

## Conclusions

We discuss the importance of differentiating device-related TMA from other TMAs, the pathophysiology of device-related TMA, and the diversity in its progression. This case highlights the importance of understanding the pathophysiology of TMA from a microscopic perspective. We believe that a general physician who can manage the general condition should be available to recognize the pathophysiology of TMA accurately. Therefore, it is necessary to improve the quality of general practices in community hospitals.
